# Identification of hub genes associated with severe COPD via WGCNA and immune infiltration analysis

**DOI:** 10.1038/s41598-025-18602-w

**Published:** 2025-10-06

**Authors:** Li-na Zhao, Yong Liang, Wei Yang, Xiao Yang, Hai-juan Peng, Yi-min Wang, Ya-qiang Li, Qi Zhang

**Affiliations:** 1 Department of Clinical Laboratory, Blood Transfusion Department, Zibo Central Hospital, Zibo, Shandong China; 2 Department of Research and Development, Shenzhen Xbiome Biotech Co., Ltd., Shenzhen, China; 3 Salus BioMed Company Limited, Shenzhen, Guangdong China; 4 Department of Gastroenterology, Beijing Luhe Hospital, Capital Medical University, Beijing, China

**Keywords:** COPD, Hub genes, FEV1/FVC, Biomarker, Immune infiltration, RNAseq, Respiratory tract diseases, Diagnostic markers

## Abstract

Chronic Obstructive Pulmonary Disease (COPD) is a progressive respiratory disorder characterized by persistent inflammation and airflow limitation. This study aimed to investigate immune cell infiltration patterns and identify key hub genes associated with severe COPD using integrative bioinformatics analysis. We analyzed transcriptomic data from the GSE76925 dataset, comprising lung tissue samples from 111 individuals with severe COPD (GOLD stage 3–4) and 40 healthy controls. Bioinformatic approaches included weighted gene co-expression network analysis (WGCNA), immune cell infiltration estimation via CIBERSORT, random forest classification, hierarchical clustering, and correlation with clinical parameters such as FEV1 and FEV1/FVC ratios. Our analysis revealed distinct immune infiltration patterns and identified several hub genes significantly correlated with COPD severity. Notably, FEV1/FVC remained a robust clinical marker of disease progression. The hub genes SUMO1, HMGB1, and RBM39 were found to be strongly associated with immune-related pathways and disease severity. This study highlights the value of integrating immune infiltration analysis and gene co-expression networks to better understand the pathogenesis of severe COPD. The identification of key hub genes, including *SUMO1*, *HMGB1*, and *RBM39*, provides insights into potential biomarkers and therapeutic targets for this respiratory disease. Further validation using independent cohorts and functional experiments is warranted to confirm their clinical utility.

## Introduction

Chronic Obstructive Pulmonary Disease (COPD) represents a significant global health challenge, being one of the leading causes of morbidity and mortality worldwide^1^. It is characterized by persistent airflow limitation and chronic respiratory symptoms, often resulting from prolonged exposure to harmful particles or gases, such as tobacco smoke^2^. Despite extensive research efforts, the precise molecular mechanisms governing the progression of COPD remain inadequately understood^3^.

Recent investigations indicate that immunological dysregulation, metabolic reprogramming, and alterations in gene expression are fundamental to the pathogenesis of COPD and other chronic pulmonary diseases^4^. These mechanisms exacerbate the disease’s progression, including heightened inflammation, tissue remodeling, and the deterioration of lung architecture^5^.

Elucidating the molecular underpinnings of COPD is crucial, as it may enable the development of more effective therapies and prevention strategies^6,7^. Delineating the interplay between immune cell infiltration and transcriptional pattern changes in afflicted tissues may facilitate the identification of novel treatment targets and diagnostic indicators^8^.

This research aims to examine the various biological elements influencing COPD through the integration of transcriptomic and clinical symptoms data. We will employ bioinformatics techniques to identify essential biological pathways and interactions that facilitate disease development. We will examine the patterns of immune cell infiltration and their association with local inflammatory responses in lung tissue. Concurrently, we will perform differential gene expression analysis to identify genes and pathways most closely associated with disease severity, exacerbations, and comorbidities.

Our goal is to provide deeper insights into the molecular etiology of COPD by elucidating the intricate relationships among immune responses and changes in gene expression. We aim to develop potential biomarkers for illness monitoring and therapeutic targeting, thereby improving tailored and successful treatment regimens for COPD patients.

## Experimental procedures

### Data acquisition and preprocessing

TGene expression profiles from 111 severe chronic lung patients and 40 normal controls were obtained from public database GSE76925. Clinical characteristics, including lung function indices (such as FEV1/FVC ratio and predicted FEV1 percentage), were also collected.

### Correlation analysis and random forest

Leveraging a comprehensive suite of R packages, we conducted an in-depth investigation to explore the intricate relationships among a diverse array of variables. This analysis encompassed the examination of crucial clinical metrics, including the FEV1/FVC ratio^9^, FEV1^10^, low attenuation area (LAA) at -950 HU^11^, the 15th and 10th percentiles of the attenuation histogram (Perc 15% and Perc 10%, respectively)^12^, COPD status, body mass index (BMI), smoking history (pack-years), race, and sex. By delving into the complex interplay between these multifaceted factors, we gained valuable insights into the underlying dynamics and patterns within the dataset. Subsequently, a robust random forest analysis^13^ further investigated the most significant factors associated with the development and progression of COPD, providing a deeper understanding of this complex respiratory condition.

### Clustering analysis

The samples were clustered independently based on their clinical features and gene expression patterns, resulting in the identification of groups that were predominantly composed of either healthy individuals or COPD patients. To refine the initial clustering based on clinical characteristics, outlier samples were removed.

Comprehensive cluster analysis was conducted to examine the clinical characteristics of the COPD population. This involved employing hierarchical clustering (hclust)^14^ to group participants based on their clinical profiles. Additionally, gene expression data was analyzed using weighted gene co-expression network analysis (WGCNA)^15^ and consensus clustering partition (CCP)^1,16^ methods to identify distinct gene expression-based clusters within the cohort.

### Weighted gene co-expression network analysis (WGCNA)

Weighted Gene Co-expression Network Analysis (WGCNA) was conducted using the R package WGCNA to identify gene modules that exhibit strong associations with relevant clinical characteristics. The module-trait relationships were thoroughly examined to pinpoint the modules that demonstrate robust correlations with key lung function metrics.

### Immune infiltration analysis and key gene module expression

We centered on the turquoise module, which exhibited an inverse relationship with certain clinical characteristics as revealed through the analysis conducted using Cytoscape and the STRING website. The top 23 hub genes (Table 1) within this module were further examined to investigate the hub degree-based network and the significance of these hub genes. Functional enrichment analysis, including Gene Ontology (GO) and Kyoto Encyclopedia of Genes and Genomes (KEGG) pathway analysis, was conducted using the STRING web tool to elucidate the roles of these hub genes in the pathogenesis of COPD. The enrichment results were subsequently visualized using Cytoscape to better illustrate the involved biological processes and pathways.

**Table 1:**
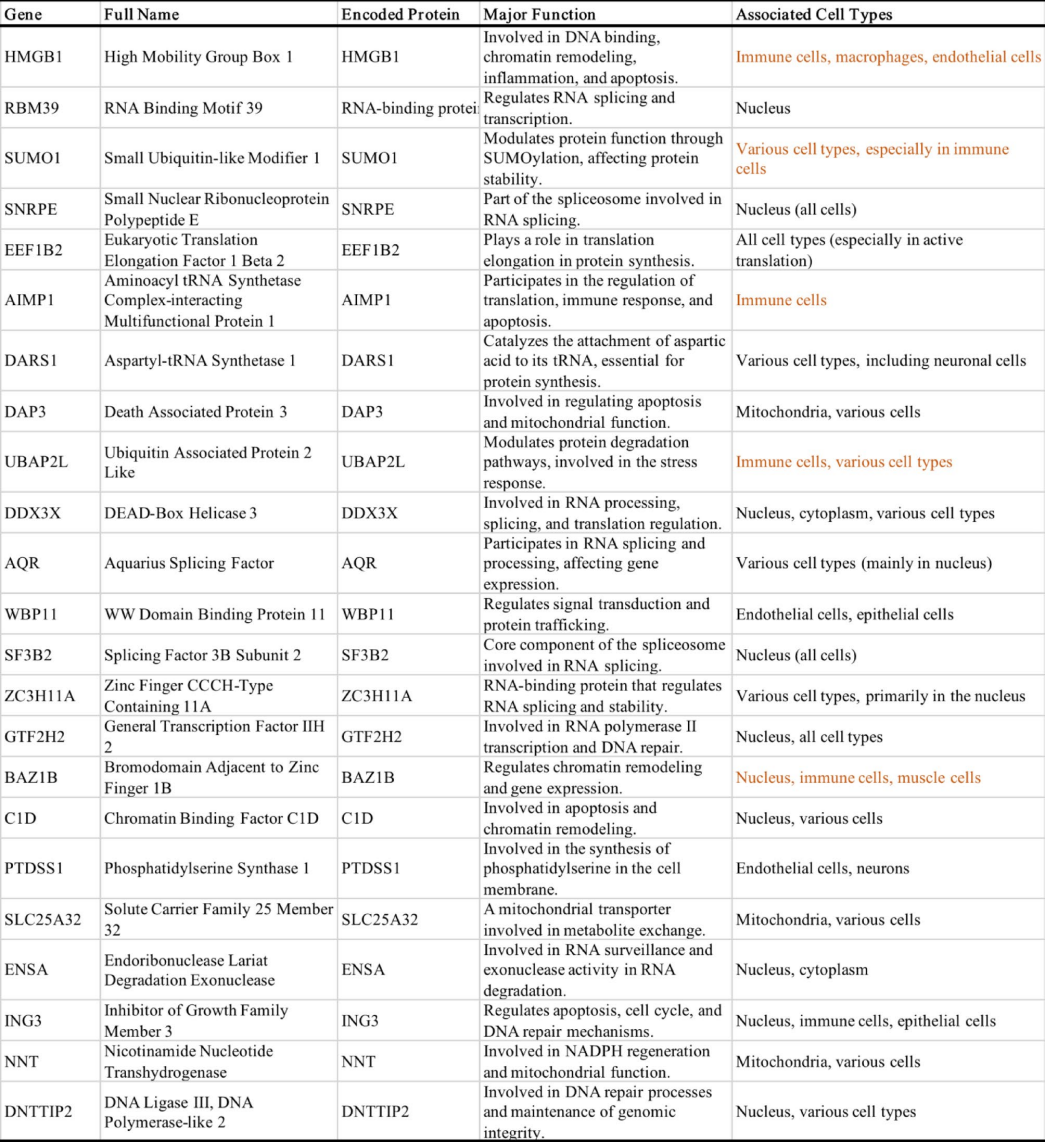
Information of 23 hub genes.

## Results

### Clinical feature correlation and clustering

The GSE76925 dataset used in this study originates from Brigham and Women’s Hospital in the United States and includes lung tissue transcriptomic data from 111 individuals with severe COPD (GOLD stage 3–4) and 40 control individuals with normal lung function. All subjects are former smokers, and the samples were obtained during lung tissue surgical resection.

**Fig. 1:**
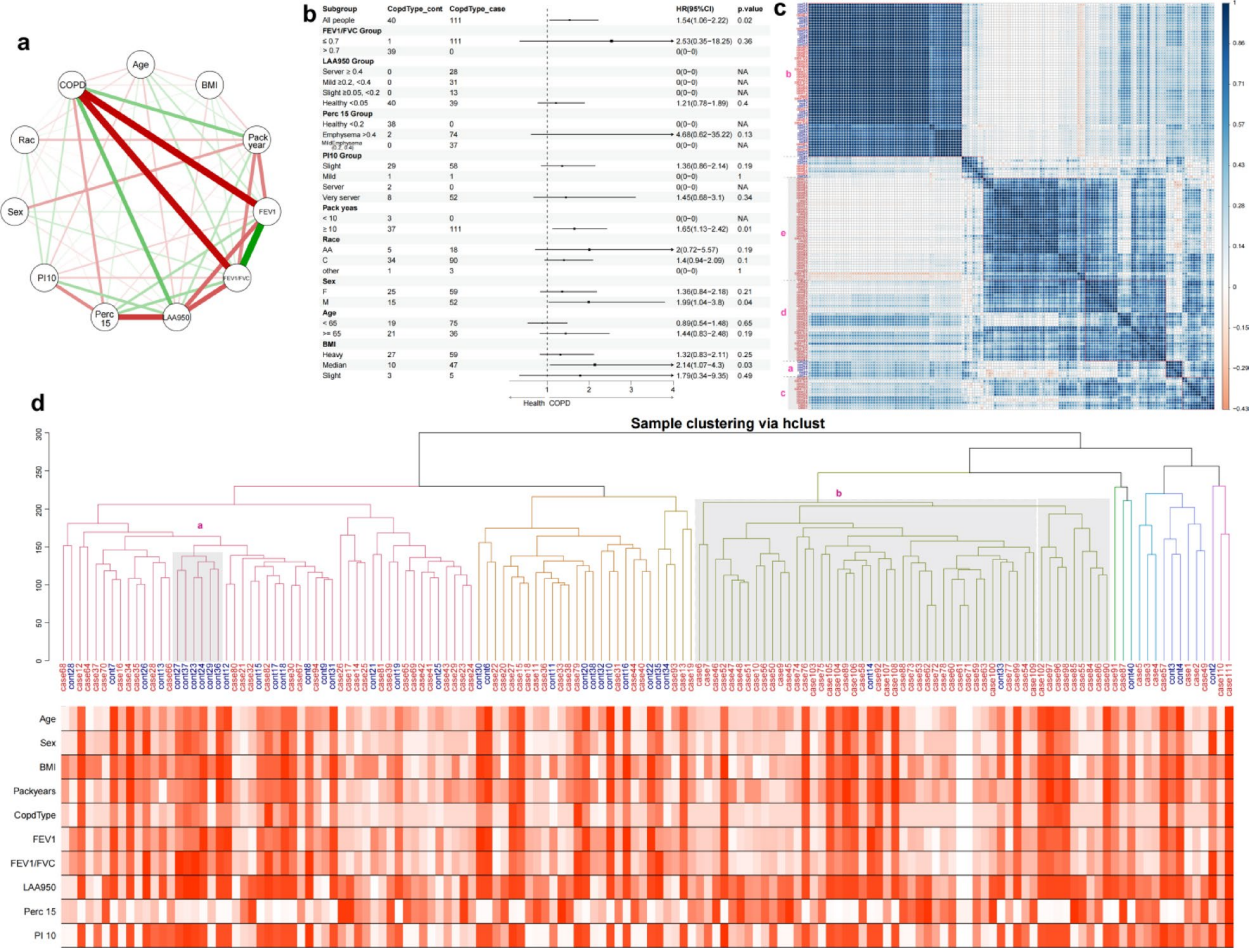
Relationship between clinical factors and COPD status. (**a**) Network plot showing correlation between clinical features and COPD status. (**b**) Random forest analysis shows those clinical factors linked to Chronic Obstructive Pulmonary Disease (COPD). (**c**) COPD patients can be divided into different groups based on their clinical profiles. Groups c, d, and e are primarily COPD patients, while Group a, and b mainly consists of healthy individuals. (**d**) Cluster analysis identifies COPD-predominant patient group based on clinical characteristics.

To elucidate the role of clinical features in patient stratification, we first analyzed the correlation strength among clinical characteristics using data from 151 samples, which included both severe chronic lung patients and normal controls. The network plot (Fig. 1a) depicting these correlations indicated a significant positive relationship among the lung function indices: the FEV1/FVC ratio, the percentage of predicted FEV1, the percentage of LAA 950, the percentage of Perc 5, the percentage of PI 10, and COPD disease status. These findings reflect the established use of FEV1/FVC and percent predicted FEV1 in the clinical diagnosis and severity grading of COPD, consistent with current guidelines.

To further investigate the impact of binary classification on patient risk factors, we employed a random forest analysis (Fig. 1b), which confirmed that FEV1/FVC ratio is the most crucial factor associated with COPD^17^. This method reinforced the importance of FEV1/FVC ratio in predicting disease severity^18^.

Subsequently, we performed a similarity-based clustering analysis using clinical characteristics (Fig. 1c). This analysis, which excluded the seven most divergent samples, revealed distinct groups. Group a predominantly included healthy controls, while Groups c, d, and e were mainly composed of COPD patients. These results suggest that clinical characteristics can partially differentiate COPD subtypes^19^. Additionally, Group b and Group f included both healthy and COPD patients, indicating that some individuals were mixed between the healthy and COPD groups.

Finally, we integrated clinical characteristics with gene expression profiles for clustering analysis (Fig. 1d). This combined approach demonstrated that clinical characteristics could distinguish disease and health states to a certain extent, with one cluster predominantly comprising COPD patients. This finding further confirms the utility of combining clinical characteristics and gene expression in identifying COPD subtypes and supports their potential role in enhancing the precision of COPD diagnosis and treatment stratification.

### Uncovering gene expression patterns in COPD using weighted gene co-expression network analysis (WGCNA)

**Fig. 2:**
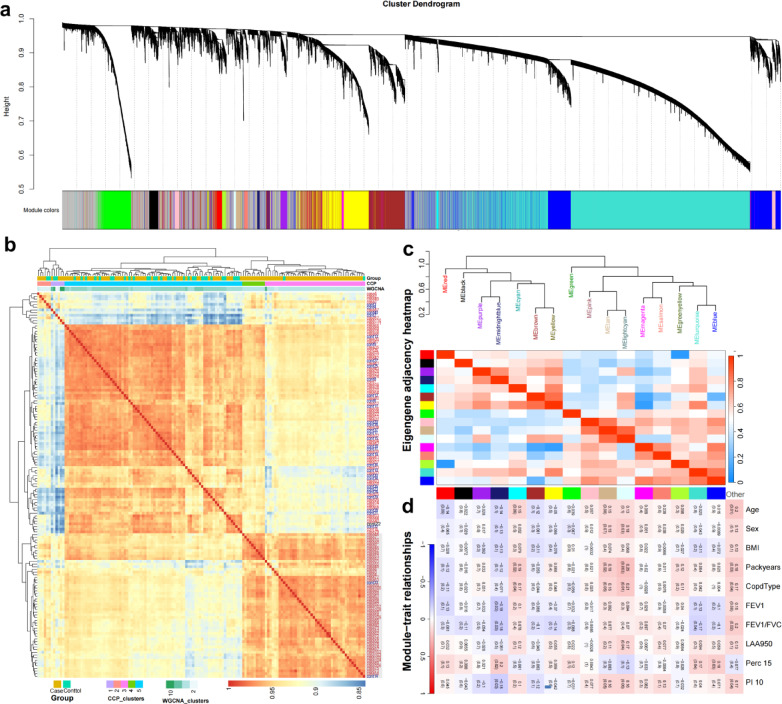
Association of gene modules with various clinical features. (**a**) This image shows a cluster dendrogram of gene co-expression modules identified using the WGCNA method. Each color represents a distinct module. (**b**) Eigengene adjacency heatmap showing sample clustering results using CCP and WGCNA, compared with sample status (case/control). (**c**), (**d**) Heatmaps show the correlations between the phenotypes and gene modules obtained from the WGCNA analysis.

To further understand the gene expression patterns associated with COPD, we performed weighted gene co-expression network analysis (WGCNA) on gene expression data. This analysis identified distinct gene co-expression modules, each represented by a different color in the cluster dendrogram (Fig. 2a).

By combining the results of WGCNA with consensus clustering partition (CCP) methods, we aimed to determine whether gene expression profiles could clearly segregate the cohort into disease and control groups, or identify well-defined disease subgroups. Both clustering methods produced consistent results, highlighting a subset of samples that consistently clustered within the COPD group, thus validating the robustness of our clustering approach (Fig. 1d; 2b).

### Correlation of gene modules with clinical phenotypes

We conducted a comprehensive correlation analysis between the identified gene modules and clinical characteristics, including age, gender, body mass index (BMI), and lung function indices. The module-trait relationships plot revealed that certain gene modules were correlated with clinical traits. Notably, the turquoise and midnightblue modules exhibited a strong negative correlation with the FEV1/FVC ratio, a critical indicator of lung function in COPD patients. This finding highlights the potential of the turquoise module genes as biomarkers for disease severity and progression in COPD (Fig. 2c, d).

These findings demonstrate the ability of Weighted Gene Co-expression Network Analysis (WGCNA) to identify gene modules that are strongly associated with clinical characteristics, offering insights into the molecular mechanisms underlying Chronic Obstructive Pulmonary Disease (COPD). The discovery that the turquoise and midnightblue modules are significantly correlated with the FEV1/FVC ratio further emphasizes the value of integrating clinical and gene expression data to enhance our understanding of COPD pathogenesis.

**Fig. 3:**
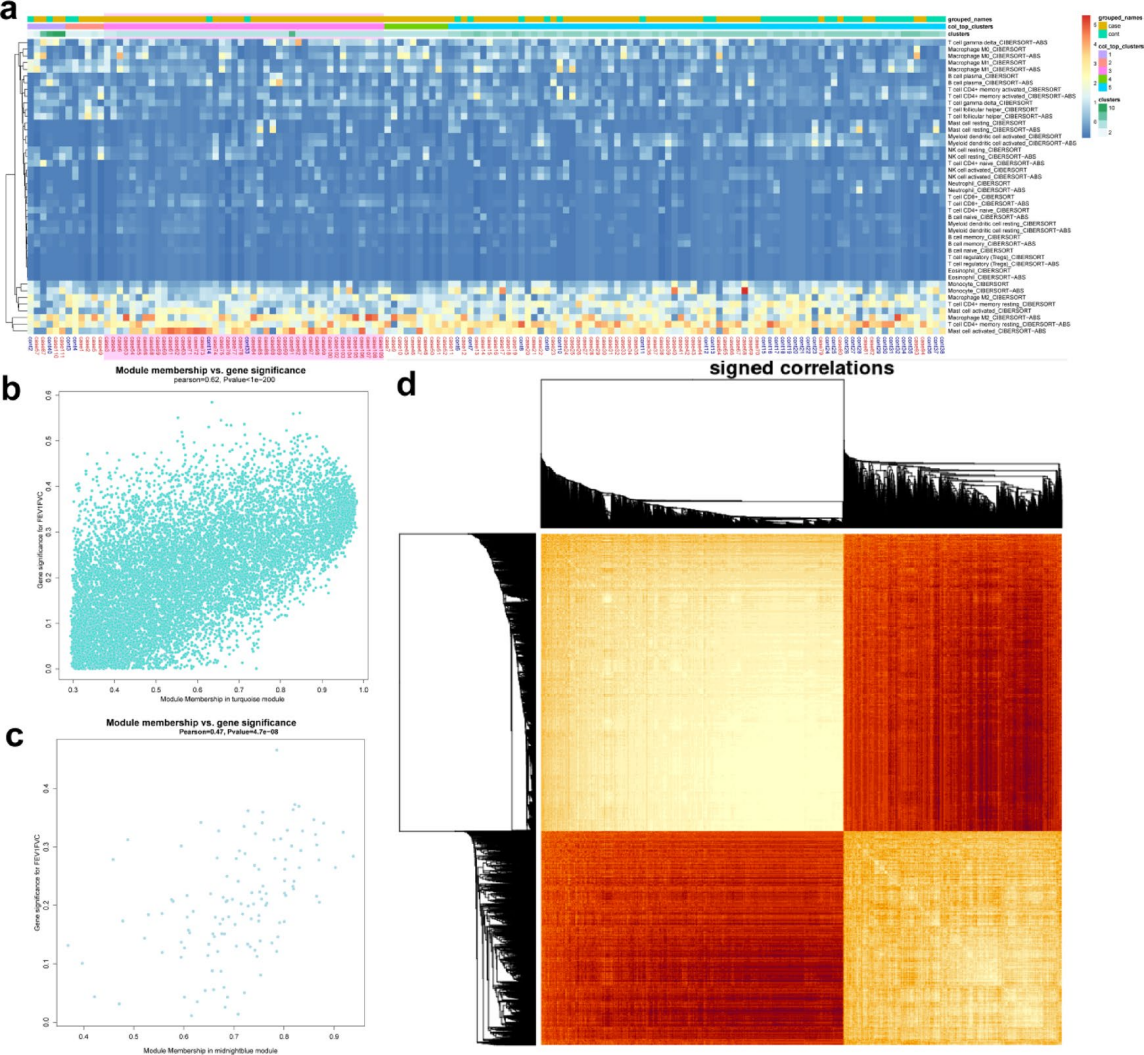
Immune infiltration of COPD. (**a**) CIBERSORT analysis evaluating the relative abundance of different immune cell subsets in the samples. (**b**), (**c**) Significance and module relationships of genes in the turquoise and midnight blue modules, strongly correlated with the FEV1/FVC ratio in WGCNA. (**d**) Strength of correlations among all genes in the turquoise module.

We examined the correlation between the FEV1/FVC ratio (FEV1F) and the highly correlated gene modules identified through Weighted Gene Co-expression Network Analysis (WGCNA) (Fig. 3b, c, d). Scatter plots revealed a strong negative correlation between FEV1/FVC and the turquoise module, which was further supported by the strength of correlations among all genes within this module (Pearson = 0.62; *p*-value < 1e-200) (Fig. 3b). Additionally, the midnightblue module showed a moderate correlation with FEV1/FVC (Pearson = 0.47; *p*-value = 4e-08). Heatmap analysis (Fig. 3d) of the 253 genes highly correlated within the turquoise module highlighted two distinct clusters of highly positively and negatively correlated genes.

### The immune infiltration in COPD

In clusters predominantly comprising COPD patients, there was a notably high proportion of activated mast cells, closely followed by M2 macrophages. This is in contrast with cluster b, where the distinction from controls was less pronounced, and the proportion of activated mast cells was moderate, accompanied by a higher presence of resting CD4 + memory T cells. In contrast cluster c, mainly composed of control samples, exhibited the highest proportion of resting CD4 + memory T cells and a lower presence of activated mast cells.

To gain deeper insights into the immune infiltration patterns in COPD, the researchers utilized the CIBERSORT algorithm to assess the relative abundance of diverse immune cell subsets across the samples (Fig. 3a). The analysis uncovered distinct profiles of immune cell composition within different clusters.

In clusters predominantly comprising COPD patients, there was a notably high proportion of activated mast cells, closely followed by M2 macrophages. This is in contrast with cluster b, where the distinction from controls was less pronounced, and the proportion of activated mast cells was moderate, accompanied by a higher presence of resting CD4 + memory T cells. Cluster C, mainly composed of control samples, exhibited the highest proportion of resting CD4 + memory T cells and a lower presence of activated mast cells.

Those findings above reveal distinct patterns of immune cell infiltration observed in samples from individuals with chronic obstructive pulmonary disease (COPD) compared to control samples. This underscores the significant association between specific immune cell subsets and the clinical manifestations of COPD. The strong correlation between the turquoise module and the ratio of forced expiratory volume in 1 s to forced vital capacity (FEV1/FVC) suggests potential molecular mechanisms underlying the involvement of immune cells in the pathogenesis of COPD. These insights warrant further investigation into these gene networks combined with infiltrating immune subtype cells as potential therapeutic targets, providing valuable clues for a deeper understanding of the molecular mechanisms underlying lung function regulation from immune cell infiltration. The findings highlight the complex interplay between the immune system and the development and progression of COPD, offering promising avenues for future research and the development of targeted interventions.

### Identification and analysis of key genes and key sub-cell type in COPD pathogenesis

**Fig. 4:**
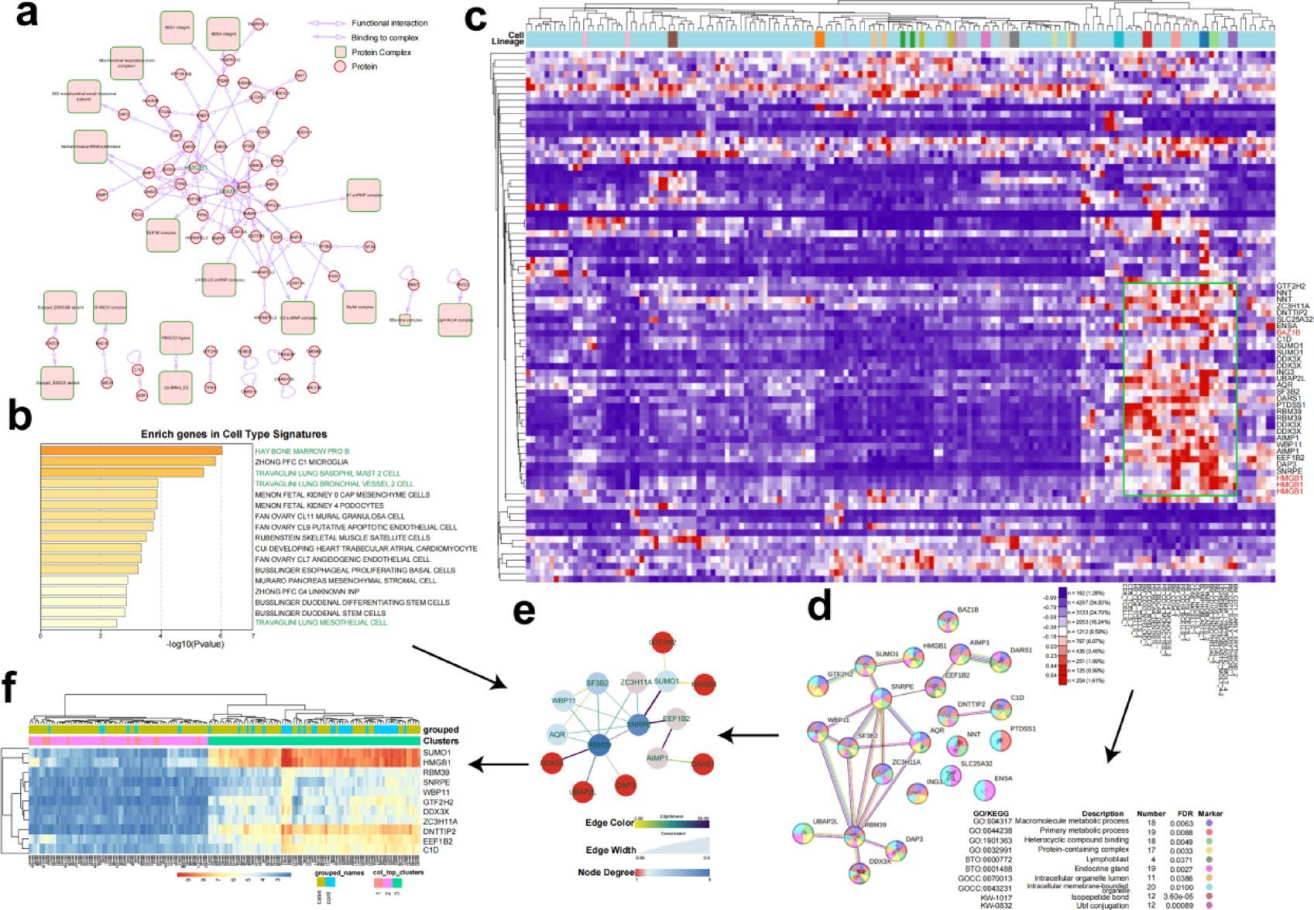
The Hub network and hub genes. (**a**) Relationship network among 253 gene-related proteins. (**b**) Enriched expression of these genes in different tissues. (**c**) Enriched expression of these genes in different cell lineages within the human body. (**d**) Protein–protein interaction network of these genes. (**e**) Key genes and central nodes. (**f**) Expression status of the key genes across all samples.

We conducted a comprehensive analysis of the highly correlated genes within the turquoise module identified through Weighted Gene Co-expression Network Analysis (WGCNA). By examining the intricate interaction networks of these 253 genes using the NDEx online platform, we uncovered a complex web of functional interactions, complex formations, and protein–protein relationships, as illustrated in Fig. 4a.

Further enrichment analysis using the GSEA Molecular Signatures Database revealed significant enrichment of these genes in key cell types implicated in COPD pathogenesis, including myeloid progenitor B cells, Travaglini lung basophil mast cells 2, Travaglini lung basophil vessel cells 2, and Travaglini lung mesothelial cells (Fig. 4b). Expression analysis across various tissue types, using the GSEA Investigate human gene sets tool (Fig. 4c), identified 23 genes with particularly high expression in myeloid and blood-related immune cell subsets.

To delve deeper into the interactions among these 23 genes, we performed protein–protein interaction (PPI)^20^ analysis (Fig. 4d) and KEGG annotation^21^ (Fig. 4d). These analyses revealed that the genes are primarily involved in pathways related to intracellular macromolecule synthesis and metabolism. Using Cytoscape^22^, we conducted MCODE topological structure analysis on the gene network (Fig. 4e), identifying a key MCODE module based on degree centrality^23^. This module contains the top 23 critical genes in the network, including *HMGB1*, and *SUMO1*^24^, two known therapeutic and monitoring targets for COPD^25–27^, and a novel hub gene, *RBM39*^28^, which was functionally related to other genes in the module (Fig. 4f).

Cluster analysis of the expression patterns of the top 23 genes across 144 samples revealed that the group with high expression of these genes was predominantly composed of control samples, while the group with low expression was mainly comprised of COPD patients with high activated mast cell infiltration. Notably, the gene *HMGB1* was significantly upregulated, indicating its potential role in the pathogenesis of COPD. Additionally, the gene *SUMO1* also showed relatively high expression, suggesting its involvement in the disease process^29^. Although *SUMO1* and *HMGB*1 were only weakly correlated, *SUMO1* exhibited a strong correlation with *SNRPE*, which in turn was strongly correlated with *RBM39*. This intricate network of gene interactions highlights the complex molecular mechanisms underlying COPD, and provides valuable insights into potential therapeutic targets and disease biomarkers.

## Discussion

The comprehensive bioinformatic analysis has yielded crucial insights into the molecular and immunological underpinnings of chronic obstructive pulmonary disease (COPD). By meticulously correlating clinical characteristics with whole-genome expression data, going beyond just differentially expressed gene (DEG) analysis, we were able to uncover additional detailed information that was missed by the DEG approach^30^. This has led to the identification of significant gene modules and key genes that hold promise as biomarkers and therapeutic targets for this debilitating respiratory condition^31^. The study’s in-depth examination of the complex interplay between genetic factors, immune responses, and clinical manifestations of COPD has provided a more holistic understanding of this prevalent and challenging respiratory disorder^32^. The findings from this comprehensive bioinformatic analysis hold the potential to inform the development of more targeted and effective interventions for managing COPD, a condition that significantly impacts the quality of life for millions of individuals worldwide^33^.

The investigation confirmed a strong positive correlation between key lung function indices, such as forced expiratory volume in 1 s (FEV1) and the ratio of FEV1 to forced vital capacity (FEV1/FVC), and the severity of chronic obstructive pulmonary disease (COPD)^34^. This finding underscores the critical association of FEV1/FVC with severe COPD and its potential as a crucial clinical marker, consistent with previous studies^3,35^. The analysis also revealed that the turquoise gene module was strongly negatively correlated with FEV1/FVC, indicating its pivotal involvement in COPD pathogenesis^36^.

Within the turquoise module, we identified 253 highly correlated genes, including the top 14 hub genes such as *SUMO1*, *HMGB1*, and *RMB39*. These genes play critical roles in immune response and inflammation pathways, positioning them as central elements in COPD pathophysiology. Notably, *SUMO*1^37–40^ with its downregulation, has been linked to reduced lung function and increased exacerbations in COPD patients^41,42^. Similarly, *HMGB1*, a key mediator of airway inflammation, has previously reported as a potential biomarker during acute exacerbations of COPD^43^, and has shown therapeutic potential in preclinical COPD models^44–47^. In addition to its extracellular inflammatory roles, *HMGB1* may also regulate RNA splicing, which could contribute to the aberrant gene expression patterns in COPD. Our findings further suggest that the downregulation of *HMGB1* in severe COPD may impair cytoplasmic immune signaling and autophagy, thereby aggravating lung inflammation and tissue remodeling. Together, *SUMO1*, *HMGB1*, and *RBM39* appear to function as an interconnected regulatory module, with significant potential to modulate and mitigate the core pathological mechanisms driving COPD development and progression. This protein triad could serve as a promising therapeutic target for addressing the complex and multifaceted nature of this debilitating respiratory condition.

While there are currently no direct therapies specifically targeting the genes *SUMO1*, *HMGB1*, or *RMB39* for the treatment of chronic obstructive pulmonary disease (COPD), ongoing research is actively exploring their potential as promising drug targets. The *SUMO1*^24^ gene, which is normally upregulated in healthy individuals^48,49^, plays a crucial role in regulating inflammatory pathways and maintaining protein stability^50^. In COPD, the downregulation of *SUMO1* may impair the resolution of inflammation, leading to chronic airway obstruction and tissue damage^27,51^. Restoring the expression of *SUMO1* could help modulate these inflammatory pathways and potentially improve overall lung function^52^. Although no direct drugs targeting *SUMO1* have been developed yet, pharmacological agents that enhance the SUMOylation process, such as small molecule *SUMO1* activators or gene therapies, could be explored as potential treatments for COPD^53^. By restoring normal immune regulation and reducing chronic inflammation, these therapeutic approaches may offer new avenues for managing this debilitating respiratory condition^54^.

Similarly, *HMGB1*, which is upregulated in healthy individuals and downregulated in server COPD, is a key regulator of inflammation by activating immune cells, and tissue damage in COPD^55^. This kind of *HMGB1* that works is mostly the function of *HMGB1* in the cytoplasm^56^. Cytoplasmic *HMGB1* promotes autophagy in cells in response to various environmental stresses, including starvation and oxidative damage^57,58^. Another important function of cytoplasmic *HMGB1* is to form complexes with free nucleic acids, thereby activating the TLR and cGAS-STING pathways of innate immunity and promoting nucleic acid-mediated innate immune responses^59^. That is, the reduction of *HMGB1* in the cytoplasm in server COPD may indicate an impaired ability to regulate the inflammatory response in the lungs, contributing to ongoing tissue remodeling and airway destruction^60,61^.

The *RBM39* gene, which regulates RNA processing and splicing, is another key gene that is downregulated in severe COPD^62,63^. In normal conditions, *RBM39* ensures the proper splicing of mRNA, helping maintain normal cellular functions^63^. However, its dysregulation in COPD may result in defective protein production and contribute to cellular dysfunction. Upregulating *RBM39* could potentially restore normal RNA splicing^64^ and protein expression, providing a new strategy for treating COPD. While specific drugs targeting *RBM39* are not yet available, the development of small moleculars or RNA-based therapies to modulate splicing factors could provide a novel approach to restoring gene expression in COPD.

Apart from the related gene modules, further analysis of immune cell compositions revealed distinct immune profiles between COPD and control groups. Consistent with previous findings, we observed elevated proportions of M2 macrophages^65^ and activated mast cells^66–68^ in severe COPD patients, highlighting the immune dysregulation and chronic inflammatory state underlying disease progression. Notably, a recent bioinformatics study investigating COPD-associated pulmonary hypertension (COPD-PH) also emphasized the interplay between hypoxia-inducible genes and immune-mediated inflammatory cytokines in modulating COPD pathogenesis^69^. In contrast, individuals with milder disease phenotypes closer to healthy individuals or healthy individuals showed a lower prevalence of these immune cells, indicating a less pronounced inflammatory response^70^. These findings collectively suggest that immune cell activation and hypoxia-associated signaling co-contribute to the pathophysiological remodeling observed in COPD lungs.

M2 macrophages, known for their anti-inflammatory and tissue repair functions, may contribute to a maladaptive immune response in chronic obstructive pulmonary disease (COPD), promoting fibrosis and exacerbating airway remodeling^71^. Meanwhile, activated mast cells are key players in the allergic inflammatory environment in the lungs^72^.

In contrast, healthy individuals exhibited relatively low proportions of these immune cell subsets, reinforcing the notion that the expansion of M2 macrophages and mast cells in COPD is a hallmark of disease severity and progression, highlighting the abnormal immune activation in COPD^73^. Interestingly, resting memory T cells were found in relatively moderate to slightly higher proportions in healthy controls compared to severe COPD patients^74^. These T cells, which are critical for long-term immunity, typically help maintain immune homeostasis and are involved in the resolution of inflammation^75^. However, in COPD, the shift away from a balanced immune profile toward an increased presence of M2 macrophages and mast cells suggests an immune dysregulation that contributes to chronic inflammation and lung tissue damage^76^.

Given their crucial role in disease pathogenesis, targeting M2 macrophages and mast cells presents a potential therapeutic strategy for COPD^77^. Therapies designed to inhibit the activated or accumulation of these cells could help reduce inflammation and tissue damage, potentially improving patient outcomes. Similarly, targeting mast cell degranulation or blocking mast cell-derived mediators may provide an avenue for controlling the excessive inflammatory response observed in sever COPD.

Recent advancements in COPD treatment have underscored the critical importance of targeting immune pathways to modulate chronic inflammation, a hallmark of this debilitating respiratory condition. The phase 3 success of Dupixent^78^, an interleukin-4 receptor antibody, has provided compelling evidence for the efficacy of this targeted immunomodulatory approach. This success highlights the immense potential of modulating the immune response to reduce the chronic inflammation and immune dysregulation central to COPD pathogenesis, offering new hope for patients.

Building on these clinical advances, our immune infiltration analysis using CIBERSORT revealed a distinct shift toward innate immune activation in severe COPD, characterized by increased M2 macrophages and mast cells, along with a notable reduction in resting memory T cells. This altered immune cell composition is consistent with recent single-cell RNA sequencing data from COPD patients, which identified dysregulated peripheral immune subsets and a decline in memory T cell populations^79^. These immune perturbations may reflect systemic immune exhaustion and chronic inflammatory signaling that extend beyond the lung microenvironment, potentially contributing to the persistent inflammatory state characteristic of severe COPD.

After that, restoring or upregulating *SUMO1* could help re-establish immune homeostasis and reduce inflammation. Additionally, its role in T cell regulation suggests that it may help modulate the immune response, potentially controlling the maladaptive immune activation observed in COPD^80^.

## Conclusion

The identification of key hub genes and immune cell populations in chronic obstructive pulmonary disease (COPD) provides a foundation for developing targeted therapies that address the core mechanisms of the disease. By targeting both the molecular pathways and the immune cell subsets involved in COPD pathogenesis, we can create more personalized and effective treatment strategies. These therapies hold the potential to reduce inflammation, improve lung function, and slow disease progression, ultimately enhancing the quality of life for COPD patients^36^.

## Data Availability

The data that support the findings of this study have been obtain from NCBI GSE76925 (https://www.ncbi.nlm.nih.gov/geo/query/acc.cgi?acc=GSE76925).
